# Pure Uterine Lipoma and Focal Nodular Hyperplasia of the Liver: Concurrence of a Rare Tumor and Another Incidental Finding

**DOI:** 10.1155/2020/8891820

**Published:** 2020-11-01

**Authors:** Mohammad Hossein Anbardar, Neda Soleimani, Seyed Ali Malek-Hosseini, Shirin Moradmand

**Affiliations:** ^1^Department of Pathology, Shiraz Medical School, Shiraz University of Medical Sciences, Shiraz, Iran; ^2^Department of Pathology, Shiraz Transplant Center, Abu Ali Sina Hospital, Shiraz University of Medical Sciences, Shiraz, Iran; ^3^Department of Surgery, Head of Organ Transplant Unit, Shiraz University of Medical Sciences, Shiraz, Iran; ^4^Department of Surgery, Shiraz Transplant Center, Abu Ali Sina Hospital, Shiraz University of Medical Sciences, Shiraz, Iran

## Abstract

**Background:**

Fatty uterine tumors, especially pure uterine lipoma, are very rare, but clinically and radiologically, they can mimic other primary benign and malignant uterine neoplasms. *Case Report*. A multipara 61-year-old postmenopausal woman presented with abnormal vaginal bleeding for 3 months. Assessment by ultrasound and magnetic resonance imaging (MRI) revealed a hyperechoic mass in the uterine corpus. Furthermore, during radiologic investigation, there was an incidental isoechoic mass in the left lobe of the liver. Pure uterine lipoma and hepatic focal nodular hyperplasia were diagnosed.

**Conclusion:**

Pure lipoma of the uterus, even though rare, has to be kept in the differential diagnosis of uterine neoplasms, especially in postmenopausal women. Till now, just a few concurrent tumors have been reported with pure uterine lipoma, and among them, FNH is the first extragenital tumor.

## 1. Introduction

The incidence of uterine fatty tumors varies from 0.03 to 0.2%. This includes mixed lipoma and pure lipoma of the uterus. Pure uterine lipomas are very rare, and only few cases have been reported in literature. Both clinically and radiologically, they can mimic other primary benign and malignant uterine neoplasms [1]. We hereby present a postmenopausal woman with concurrent uterine and liver mass, pure lipoma, and focal nodular hyperplasia (FNH), respectively. This is the first reported extragenital concurrent tumor with pure uterine lipoma.

## 2. Case Report

**Figure 1:**
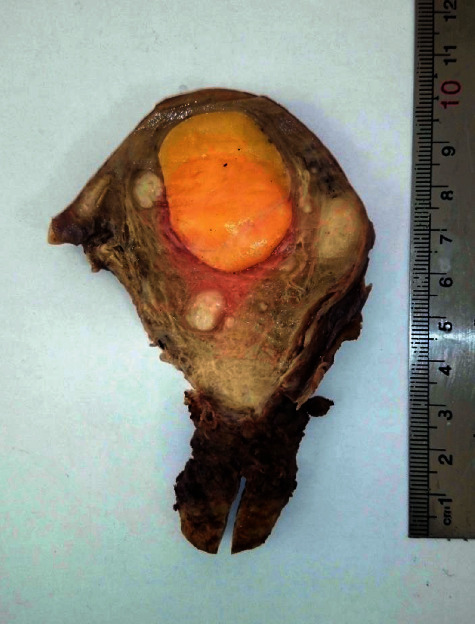
Cut section of the uterus showing an intramural well-defined bright yellow homogenous mass.

**Figure 2:**
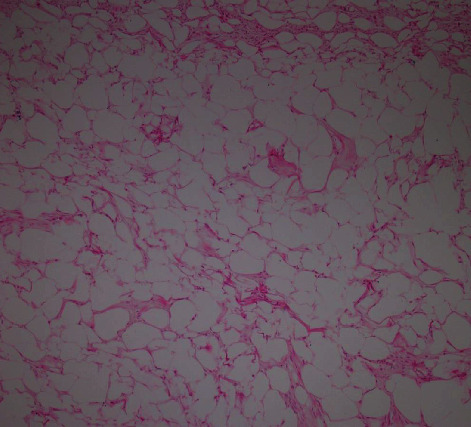
Mature white adipose tissue with bundles of smooth muscle at periphery (×40, H&E).

A multipara 61-year-old postmenopausal woman presented with abnormal vaginal bleeding for 3 months. There was no associated pain or weight loss. She experienced the last menstruation at the age of 53 years. She had regular menstruation from menarche to menopause and never took hormonal replacement therapy. The patient was a known diabetic on oral medication (metformin) with no previous history of surgery and no family history of malignancy. Physical and pelvic examination did not show any abnormality. No abnormalities were observed in the laboratory findings including liver function, hepatitis B surface antigen, hepatitis C antibody, and tumor markers (CEA, CA19-9, and AFP). Cervical cytology was normal, too. Assessment by ultrasound and magnetic resonance imaging (MRI) revealed a hyperechoic mass in the uterine corpus. Furthermore, during radiologic investigation, there was an incidental isoechoic mass in the left lobe of the liver. The patient underwent total abdominal hysterectomy (TAH), right-sided salpingo-oophorectomy, and left lateral liver segmentectomy. During gross pathologic evaluation, dissection of the uterine corpus showed an intramural well-demarcated mass with a greasy yellow cut surface measuring 4 × 4 × 4 cm (Figure 1). On microscopic examination, the mass was entirely composed of mature adipose tissue with bundles of smooth muscle at periphery, and the histology confirmed pure uterine lipoma (Figure 2). Endometrium was atrophic, and the right adnexa showed no pathologic abnormality.

**Figure 3:**
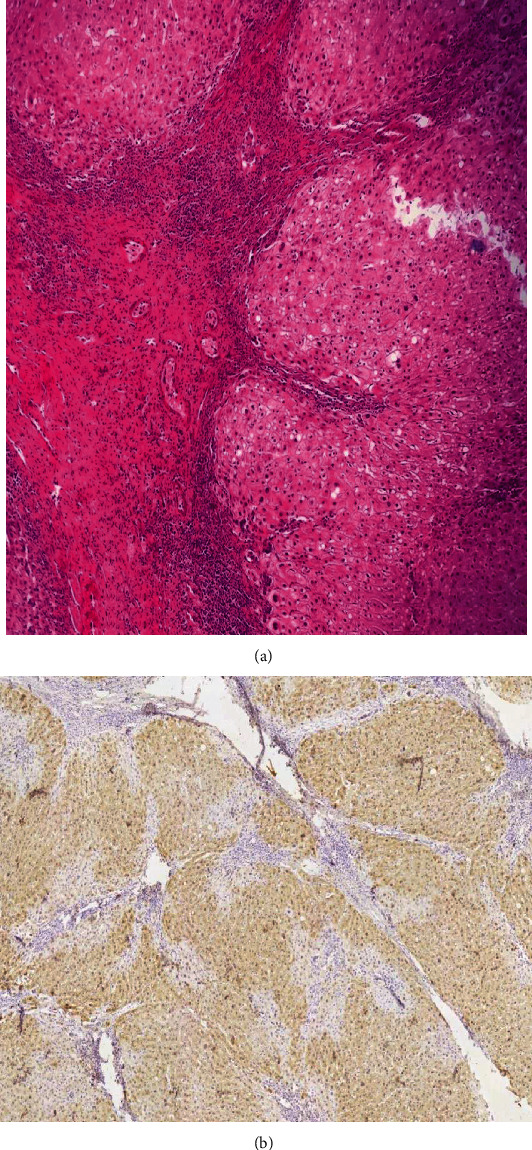
(a) Bland hepatocytes surrounded by fibrous septa that contain artery branches and variable degree of ductular reaction (×100, H&E). (b) Immunohistochemistry study for glutamine synthetase (GS) showing a map-like pattern of positivity in hepatocytes (×40).

Although the radiologic and pathologic findings of the liver mass were in favor of a classic FNH, measuring 8 × 5 × 4 cm, immunohistochemistry (IHC) was done for complete ruling out of hepatocellular adenoma. Glutamine synthetase (GS) stained the hepatocytes in a map-like pattern and confirmed the diagnosis of FNH (Figure 3).

## 3. Discussion

Fatty tumors account for about 0.03-0.2% of all uterine tumors. Although lipomas are very common soft tissue tumors, pure uterine lipomas are extremely rare and only a few cases have been reported in medical literature. They have to be differentiated from more common mixed lipomatous tumors such as lipofibroma, lipoleiomyoma, and fibromyolipoma [1–4].

The clinical manifestations do not usually differ greatly from those caused by leiomyomas of comparable size, except that they affect women that are somewhat older and normally postmenopausal. Some patients present with symptoms such as pelvic pain, discomfort, and vaginal bleeding.

The most common location is the uterine corpus. The majority are intramural; however, they can be found anywhere in the uterus or cervix and can be subserosal or submucosal. They are usually single but could be multiple as well. The size ranges from few millimeters to as large as 32 cm. Concomitant uterine leiomyoma is commonly found, although this is not present in all patients. Their prognosis is excellent [5, 6].

Ultrasonography and computed tomography finding may be nonspecific. Although magnetic resonance imaging (MRI) can be useful in identifying the fatty nature of the lesion preoperatively, most of the cases are diagnosed postoperatively on histopathological examination. MRI also highlights internal components and differentiates these lesions from adnexal masses such as benign cystic ovarian teratoma, malignant degeneration of cystic teratoma, nonteratomatous lipomatous ovarian tumor, pelvic lipoma, and liposarcoma and very rare lipomatous tumors of the uterus: angiomyolipoma of the uterus, fibromyolipoma of the uterus, and myelolipoma of the uterus [6, 7].

Microscopically, the diagnosis of pure lipoma should only be made when smooth muscle, if any, is confined to the periphery of the tumor [1]. There is no myometrial invasion.

The pathogenesis of these lesions in the uterine wall continues to be an enigma. It is not really known how a fatty tumor can develop where fatty tissue did not previously exist under normal conditions. Despite some raised theories, including metaplasia of the smooth muscles or cells of the connective tissue, fatty infiltration or degeneration of connective tissue, proliferation of perivascular fat cells, or misplaced embryonic fat cells, the exact pathogenesis is still unclear [8, 9].

Uterine lipoma can mimic a variety of other uterine neoplasms including malignancy. They might be misdiagnosed as sarcomas due to the old age of the patients, rapid progression of abdominal swelling, abdominal pain, and the well-circumscribed hyperechoic texture on ultrasonography [10].

Certain associations have been observed between uterine lipomas and cervical cancer, thecoma, struma ovarii, Brenner tumor, endometrial polyps, and more rarely with endometrial carcinoma. The role that these lesions may play in the genesis of endometrial neoplasia is not well known although some authors point to a hypothetical androgen to estrogen conversion in the fat of the lipoma [7, 11–14]. Our case presented with pure uterine lipoma and FNH. FNH is a benign sex hormone-related hepatic tumor that usually involves females of childbearing age. The patient was a 61 y/o postmenopausal lady, and concurrence of uterine lipoma and FNH reinforces the hypothesis of sex hormone conversion by lipoma. FNH is the first reported coincidental tumor with pure uterine lipoma beyond the female genital tract.

## 4. Conclusion

Pure lipoma of the uterus, even though rare, has to be kept in the differential diagnosis of uterine neoplasms, especially in postmenopausal women, but the presence of a hyperechoic uterine tumor in a postmenopausal woman raises the possibility of malignancy, especially when there is another synchronous large mass, and just histopathologic assessment can prove otherwise. Till now, just a few concurrent tumors have been reported with pure uterine lipoma, and among them, FNH is the first extragenital tumor.

## Data Availability

All supporting data (macroscopic and microscopic imaging) are included in the manuscript.
